# A Practical Treatise on the Diseases of the Testis, and of the Spermatic Cord and Scrotum

**Published:** 1844-01

**Authors:** 


### Art. II.

*A Practical Treatise on the Diseases of the Testis, and of the Spermatic Cord and Scrotum.* By T. B. Curling.—*London*, 1843. 8vo, pp. 542. With Illustrations.

This is not a book written to order, to satisfy the necessity for printing periodically experienced by the brethren of the Row; but is, we find from the author's preface, the legitimate and spontaneous issue of personal observation and research, aided by ample opportunities and abundant experience.

"My attention having been directed in the year 1835 to the subject of the morbid anatomy of the testis, I have since lost no opportunity of studying the pathological changes to which this organ is liable. My inquiries have been much facilitated by a connexion formed very early in professional life with a large hospital and with a dispensary, which have supplied me with abundant means of acquiring a practical knowledge of the diseases of this important organ. The result of these investigations having furnished facts which appear of some interest and value in relation to certain affections of the testis, but imperfectly understood, and to the treatment generally of the disorders of this part, I have ventured to submit them to the consideration of my professional brethren." (Pref. p. i.)

In addition to the practical improvements thus announced, the author's "Researches on the structure of the testis," have led him "to describe certain parts rather differently from other anatomists," and have enabled him "to throw some light on the interesting subject of the descent of the testis." (Pref. p. ii.) In fact, the present work does not appear as a mere digest of the labours and experience of others, but avowedly professes to augment our anatomical, physiological, pathological, and practical knowledge respecting the organs and the diseases it treats of, and it is our duty to present such an analysis of it to our readers as may enable them to judge of its general character and particular merits.

The First Part of Mr. Curling's work is devoted to the anatomy of the scrotum and testis, and to some considerations on the functions of the latter organ. It would be tedious as well as unnecessary to examine this portion of the book in detail, and we shall merely advert to a few points that seem to require notice.

*The scrotum.* There is considerable difference of opinion respecting the anatomy of the parts intervening between the skin and the tunica vaginalis. Winslow and his successors described them as consisting of—a very delicate layer of cellular membrane; the dartos; a tolerably thick layer of cellular membrane; and the cremaster muscle Many, perhaps most modern anatomists, for example, Blandin and Cruveilhier, consider that the dartos alone intervenes between the skin and the cremaster, but recognize between the cremaster and the tunica vaginalis, the common fibrous sheath or tunic of the cord and testicle, derived from or continuous with the transversalis fascia. Sir A. Cooper, though he does not mention the last structure, and denies the existence of the dartos, nevertheless agrees with those authors as to the number of parts that may be discriminated by dissection between the skin and the tunica vaginalis; and he states these to be the cellular tissue of the scrotum, the superficial fascia of the cord, and the cremaster muscle. Velpeau adopts a kind of middle term between these last two views, and enumerates the dartos, the superficial fascia of the cord, the cremaster, and the common tunic of the cord and testicle. We might readily extend the list of conflicting authorities, but we believe we have noticed those that are most important,—enough at least for our purpose. As to the structure and connexions of the various tissues of the scrotum, we need only notice the various opinions that have been advanced respecting the dartos. Winslow considered it a true muscle, Cruveilhier thinks it a peculiar contractile tissue intermediate between cellular membrane and muscular fibre. Boyer seems to regard it as a fibrous membrane; Meckel and Sir A. Cooper say it is mere cellular tissue, or in other words deny its existence as a distinct and independent structure; and Velpeau ingeniously reconciles these discrepancies by saying, that it is indeed cellular membrane, which is however occasionally transformed into true muscle. Some, with Cruveilhier, consider the dartos as single; others describe it as being double, but, so far as we know, all who admit its existence have hitherto agreed that it forms the septum scroti. As to its extent and attachments, some say it is continuous with the superficial fascia, others that it is connected with the aponeurotic structures of the penis and of the perineum; and others again describe it as attached to the rami of the os pubis and ischium.

Mr. Curling's account of the anatomy of the scrotum is somewhat different from any of those which we have thus briefly passed in review. He describes the subcutaneous layers of the scrotum as consisting of the dartos, a large quantity of cellular tissue, the superficial spermatic fascia, and the cremaster muscle. We do not think that this description is accurate. In the first place, as our author admits the existence of the dartos as a separate structure, we do not know where he finds "the large quantity of cellular tissue" between it and the superficial fascia of the cord, for the only *reticular* tissue that exists in the scrotum in *large quantity,* is that which is known as the dartos. A second error, as we think, and a more important one, consists in the omission of any mention of the deep fascia of the cord or common envelope of the cord and testicle, an omission into which Mr. Curling has probably been led by the authority of Sir A. Cooper. We notice this not merely because it is an anatomical oversight, but because the structure in question is of some pathological importance, inasmuch as it is frequently the seat of encysted hydrocele of the spermatic cord, in the adult at least. We may just observe, that Mr. Curling says nothing respecting the extent or the attachments of the dartos. He mentions Mr. Bowman's opinion, that it is muscular and composed of "unstriped elementary fibres," and says, we think erroneously, that the septum scroti is formed, not by the dartos, but by the cellular membrane beneath it.

We are not exactly certain whether Mr. Curling claims originality in his views respecting the cremaster muscle, though his language leads us to suspect that he does. He says, (p. 5,) "this muscle is usually considered and described as a part or process of the *oblique internus abdominis.* It has, however, separate attachments; and its office and connexions are so entirely distinct, that it ought to be regarded as an independent muscle." And at p. 31, after again alluding to M. J. Cloquet's view that the cremaster muscle is formed by the testicle drawing down, during its descent, the inferior fibres of the lesser oblique muscle, he says, "this view is, as I have shown elsewhere, clearly erroneous and inaccurate." Now, the refutation of Cloquet's view has not, as this language implies, been reserved for our author; its inaccuracy has been shown long since. Cruveilhier, for example, has expressly contradicted it. (Anatomie Descriptive, t. ii, pp. 58 and 727.) Mr. Curling's description of this muscle is precisely similar to that of Sir A. Cooper, save that Sir A. Cooper describes its internal origin as attached, on the inner side of the abdominal ring, to the lower part of the sheath of the rectus muscle, while our author describes it as attached to the os pubis, as well as to the sheath of the rectus; but the internal attachment of the cremaster to the os pubis is distinctly mentioned by Scarpa and others.

*The testis.* The description of the testicle is clear, copious, and accurate; and embodies the researches of Krause, Lauth, Müller, and Gulliver, respecting the minute anatomy of the secreting structure of this organ. In a note (p. 8) it is said, from a misprint no doubt, that Cruveilhier estimates the thickness of the testicle at three lines, it should be eight lines. Meckel states the average weight of the testicle to be four drachms, and Sir A. Cooper about an ounce. Mr. Curling says, "I have found the mean of these two estimates, viz. six drachms to be the ordinary weight of the sound testis of a healthy adult." (p. 9.)

*Descent of the testicle.* Mr. Curling has advanced some new views respecting this very interesting and obscure subject, which we shall allow him to explain in his own words. After describing the situation of the testicle in the fœtus and the general configuration of the gubernaculum testis, he proceeds thus:

"The lower part of this process (the gubernaculum) passes out of the abdomen at the abdominal ring, and diminishing in substance and spreading, terminates in three processes, each of which has a distinct attachment. The central part and bulk of the gubernaculum is composed of a soft, transparent, gelatinous substance, which, on examination in the microscope, is found to consist of nucleated cells, the primitive cellular tissue: this central mass is surrounded by a layer of well-developed muscular fibres, which may be distinguished by the naked eye, and which can be very distinctly recognized in the microscope to be composed of 'striped elementary fibres.' These muscular fibres, which may be traced the whole way from the ring to the testis, are surrounded by a layer of the soft elements of the cellular tissue, similar to that composing the central mass; and in the same way as the testis the whole process, except at its posterior part, is invested with peritoneum. On carefully laying open the inguinal canal, and gently drawing up the gubernaculum, the muscular fibres may be traced to the three processes, which are attached as follows: the external and broadest is connected to Poupart's ligament in the inguinal canal; the middle forms a lengthened band, which escapes at the external abdominal ring, and descends to the bottom of the scrotum, where it joins the dartos; the internal passes in the direction inwards, and has a firm attachment to the os pubis and sheath of the rectus muscle. Besides these, a number of muscular fibres are reflected from the internal oblique on the front of the gubernaculum. It thus appears that the attachments of the muscle of the gubernaculum, and those of the cremaster in the adult are exactly similar. I have succeeded in tracing out the former before the testis has descended, at different stages of the process, and immediately after its completion; and of the identity of the two no doubt can be entertained. (pp. 30-1.) ..... In the passage of the testis from the abdomen to the bottom of the scrotum, the gubernaculum, including its peritoneal investment and muscular fibres, undergoes the same change as that which takes place in certain of the *rodentia* at the access of the season of sexual excitement; the muscle of the testis is gradually everted, until, when the transition is completed, it forms a muscular envelope, external to the process of peritoneum, which surrounds the gland and front of the cord. As the testis approaches the bottom of the scrotum, the gubernaculum diminishes in size, owing to a change in the disposition of its cellular elements; the muscular fibres, however, undergo little or no diminution, and are very distinct around the tunica vaginalis in the recently descended testis. (p. 33.)......the middle attachment of gubernaculum, which may be traced to the dartos at the bottom of the scrotum, gradually wastes away, and soon becomes indistinct, though slight traces of this process often remain to the latest period of life. Thus, after death, in dragging the testis of an adult out of the scrotum by pulling the cord, the lower part of the gland, which is uncovered by serous membrane, is often found connected to the bottom of the scrotum by a band of firm and dense cellular tissue......This band is the remains of the middle attachment of the gubernaculum. In cases in which the testis has been retained in the groin, I have traced a cord of dense tissue from the gland to the lower part of the scrotum." (pp. 33-4.)

Mr. Curling then proceeds to deduce an explanation of the descent of the testicle.

"Hunter, Meckel, and others," he observes, "came to the conclusion that the muscular fibres of the cremaster are insufficient to bring the testis lower down than the abdominal ring, and complete the descent. They were not, however, acquainted with the attachment of this muscle to the pubis external to the ring, or it would be difficult to understand why Mr. Hunter, after arriving at the conviction that the cremaster passes up to the testis whilst in the abdomen, chiefly from analogy, was not induced by the same process of reasoning to conclude, that a muscle capable of drawing down the testicle in animals would be adequate to accomplish the same purpose in the fœtus. The necessity for some active agent to effect this change in the latter would appear to be greater even than in animals, since, in the usual position of the fœtus in utero, the passage of the testis is contrary to gravitation, and unaided by the movements of respiration. Now, when we consider the attachments and connexions of this muscle in the fœtus, the perfect condition of its fibres as ascertained by microscopical examination, and the circumstance that there are no other means, no other motive powers by which this change can be effected, or in any way promoted, I think there is no reason to doubt that the cremaster executes the same office in the human embryo, as that which it undoubtedly performs in certain animals at a particular season. The fibres proceeding from Poupart's ligament and the obliquus internus tend to guide the gland into the inguinal canal, those attached to the os pubis to draw it below the abdominal ring, and the process descending to the scrotum to direct it to its final destination. As the descent approaches completion, the muscular fibres which perform so important a part in it gradually become everted; and, instead of drawing down the testicle, acquire the new functions of elevating, supporting, and compressing it." (pp. 35-6.)

This account of the structure and attachments of the gubernaculum testis explains the descent of the testis in a very ingenious and satisfactory manner. The great difficulty has always been to understand how the testicle was conveyed from the external abdominal ring into the scrotum; for though its descent to the ring might be referred, either to the slow contraction of the gubernaculum itself, or to the action of the muscular fibres that exist on the anterior surface of that organ, the latter of these influences could clearly carry it no further than the ring, and neither the prolongation of the gubernaculum to the bottom of the scrotum, nor its muscular structure internally were suspected, until discovered by Mr. Curling, to whom the greater credit is due as the structure of these parts and the descent of the testicle have been anxiously investigated by the most eminent anatomists.

We pass over the sections on the "*spermatic fluid*" and on the "*functions of the testis;*" the latter of which, at least, might have been omitted, as everything of practical importance connected with the subject is fully discussed in other parts of the work.

Part Second treats of *Diseases of the testis*, and the first chapter is devoted to the consideration of *Congenital imperfections and malformations*, under the several heads of *Numerical excesses and defects, Deficiencies and imperfections of the vas deferens*, and *Imperfect descent of the testis;* to which last subject we shall confine our observations on this chapter.

Mr. Curling is of opinion, from his own observations, "that if the descent (of the testicle) does not take place within a twelvemonth after birth, it is rarely or never afterwards fully and perfectly completed without being accompanied with rupture." (p. 68.) This opinion has been entertained by several authors, and when we look to the mechanism of the process it might be anticipated that a late descent of the testicle must be always followed by rupture, but it is a curious fact that this is by no means uniformly the case, even when the descent is postponed to a very late period. Malgaigne, for example, (Anat. Chirurg., t. ii. p. 266,) mentions the case of a boy, aged thirteen, who being thrown from a height of seven feet, the left testicle suddenly descended from the abdomen, but was not followed by a rupture.

Mr. Curling considers at some length the causes "of a failure in the descent of the testis," (p. 68,) which have not, he observes, been much investigated, and could not have been satisfactorily explained, while the agency by which the descent of the organ is effected was unknown. Our author's views respecting that process, together with the facts that "there are few muscles in the human body whose development in different individuals varies in a greater degree than that of the cremaster," and that "a due supply of nervous energy is often denied to other muscles during fœtal existence, and is the cause of the deformities in the feet and other parts with which infants are often ushered into the world," lead him to conclude that "we may fairly enumerate paralysis and defective development of the cremaster amongst the causes of the imperfect descent of the testis." (p. 69.) But he admits, with preceding writers, that adhesion of the gland to a portion of intestine is an occasional cause, as is also a contracted state of the external abdominal ring, especially when the organ is retained within the inguinal canal.

*The condition of the undescended testis* is a subject of considerable importance, inasmuch as, to quote Sir A. Cooper's words, "when the testis remains within the abdomen, it makes a strong impression on the patient's mind that his virility is lessened or destroyed. In a case of this kind I have known the unfortunate subject of it commit suicide." (Obs. on the Structure and Diseases of the Testis, part i, p. 45.) It is, we believe, pretty generally known that the case alluded to by Sir A. Cooper does not stand alone, at least we have always understood that one who promised to become a distinguished ornament of our profession committed suicide, though his virility was unimpaired, in consequence of labouring under this infirmity—if infirmity indeed it can be called.

Some of the older authors, to whom we cannot at the moment refer, conceived that the detention of the testicles in the abdomen augmented the virile powers, because of the parts being nourished and comforted by the warmth of the viscera. We cannot say who first impugned this consoling doctrine, but Hunter promulgated a directly contrary opinion, and his authority led to a pretty general belief that an undescended testicle is imperfect both in its organization and its functions. Mr. Curling considers this question, and we shall endeavour to follow him as briefly as may be.

Mr. Curling observes that the only case Hunter met with in which both testicles remained in the abdomen contradicted his own doctrine, as the virility of the individual was unimpaired. He also quotes Mr. Owen's commentary on Hunter's views, in which it is argued that the retention of the testicles within the abdomen cannot necessarily impair their efficiency, inasmuch as they constantly remain in the abdomen in many animals, while according to Hunter's own observations, their continuance in the abdomen in those animals in which they naturally pass into a scrotum "is accompanied only with a difference of shape and size," which, Mr. Owen remarks, "may influence the quantity, but not necessarily the quality of the secretion." It is, however, important to ascertain not merely from analogy, but by actual examination, how far the organization of the human testicle is influenced by being retained in the abdomen. Mr. Curling says but three cases have been hitherto recorded in which the anatomical condition of a testicle situated within the abdomen is described. In the first of these (J. Cloquet, Recherches sur les Causes et l'Anat. des Hern. Abdom.,) the gland was of its natural shape and *size*. In the second, (Cooper, op. cit. p. 45,) the testicles, which were both within the abdomen, were nearly, *if not quite*, the natural size. In the third, (Bright, Guy's Hosp. Reports, vol. ii, p. 258,) the retained gland was much smaller than natural, but its structure was perfect. Mr. Curling has had an opportunity of adding a fourth case to this list; in it, the right testis of a lad named J. West, aged sixteen, "situated about an inch and a half above the internal ring, was very small, not larger than that of a child two years of age, and on cutting into it the gland presented the granular appearance usually remarked at that early period. . . . the left testis which was in the scrotum was four times the size of the right." (p. 75.) We are not aware of any other cases, besides those mentioned by Mr. Curling, in which the precise condition of the testicle has been particularly described; but we must observe that Sir A. Cooper, in his account of the case above referred to, says generally that he has examined *others*, several we must presume, in which, as in it, the testicle "was nearly of the same size as a healthy testis when deprived of its tunica vaginalis; and the seminiferous tubes were full of semen," that is to say, the organs were unquestionably fit for procreation.

It has been already observed that virility was unimpaired in the case of non-descent of both testicles seen by Hunter; but on the other hand, Mr. Wilson mentions a case in which a young man similarly affected, had never evinced any sexual passion. This, Mr. Curling observes, is, so far as he can ascertain, "a solitary case of impotency occurring under such circumstances, and when we consider how various are the defective sexual power, this single instance, and the case of West, *are scarcely sufficient* to confirm the opinion of John Hunter, or to invalidate the general conclusion that retention of the testes in the abdomen does not incapacitate them from performing their proper functions, a point on which it is obviously of great importance that surgeons should have it in their power, to give a confident and satisfactory opinion, and relieve the anxiety of parents." (pp. 75-6.)

This passage affords an example of the want of precision with which Mr. Curling sometimes writes, which occasionally renders it somewhat doubtful what his real opinion finally is. The weight of the evidence adduced, and the general tenor of Mr. Curling's reasoning on that evidence go to invalidate Hunter's opinion, yet here and there something is said that insinuates a doubt. Thus in the passage last quoted, the cases referred to are said to be *scarcely sufficient* to confirm Hunter's opinion, a phrase which implies that they are very nearly sufficient to do so. Again the author says, " in *all cases* of imperfect descent of the testis, whether the gland be arrested in the *abdomen* or groin, it is *nearly always* small in size," though in two of the only four dissections referred to, this was not the case, " and it cannot be doubted, that the natural situation of the testis is the one best adapted for the efficient performance of its function." (pp. 81-2.) Finally, however, Mr. Curling states that " the surgeon may confidently assure his patient that the detention of the testes in the abdomen is perfectly compatible with his virility, and in cases where there are no external marks of effeminacy or other grounds for suspecting impotency, and the patient is subject to erections, I should not consider the imperfection as offering any bar to marriage." (p. 82.)

We may perhaps be thought by some to have devoted too much space to the discussion of this question, but its importance we think warranted us in considering it rather fully ; especially as Mr. Curling's work, which merits, and we are satisfied must command, a very extensive circulation, though it contains all the elements for coming to a right conclusion respecting it, and ultimately inculcates that conclusion, yet does so with a degree of hesitation and doubt, which we cannot perceive any grounds for.

When a testicle is permanently arrested in the inguinal canal or in the groin, it is unquestionably in a worse predicament than when it remains within the abdomen, as in the former situation it is liable to pressure, friction, blows, or other injuries which may impede its nutrition or cause the supervention of inflammation or other disease. Hernia too very usually accompanies a late descent of the testis. For these reasons, says Mr. Curling, " and as the descent is seldom perfectly accomplished when delayed beyond the age of ten or twelve, I think it becomes a serious consideration in cases where the gland does not make its appearance till this late period, whether the well being of the patient would not be best consulted by our employing some mechanical means to prevent the escape of the organ from the abdomen." (p. 80.) We shall presently endeavour to estimate the value of this suggestion, but must first advert to the much more important case, where a hernia occurs while the testicle is within the abdomen.

Mr. Curling does not give a very definite opinion as to the treatment that should be adopted in this latter case. He first mentions an interesting case, in which a child a year old, neither of whose testicles had descended from the abdomen, had an inguinal rupture at both sides : Mr. Curling being consulted " in accordance with the usual practice, objected to the application of any truss." This advice was followed, and when the boy had attained the age of eight, the rupture on the right side had disappeared spontaneously, that on the left protruded very slightly, but there was no appearance of either testicle. Respecting this case Mr. Curling takes occasion to say,

"If it be granted that a testis situated in the abdomen is in a better position than one placed in the groin, that it is productive of less inconvenience, and exposed to fewer causes tending to impair its structure, that its subsequent descent, if it ever takes place, is frequently if not commonly attended with rupture, it must, I imagine, be admitted, that the advice usually given in these cases is unsound and injudicious. Had a different practice been adopted in the case of the boy just described, and a truss applied, I cannot think it would have contributed much more to his health and comfort, than leaving him for several years subject to all the inconveniences and dangers of an unrestrained double rupture." (p. 81.)

Mr. Curling however, as is too much his habit, clogs this opinion with qualifying expressions sufficient to insinuate distrust as to the general applicability of the practice he seems to prefer, but yet not sufficient to suggest rules for guidance in particular cases. Thus he follows up the passage last quoted, by saying " it must not however, be inferred, that the arrival of the testicle in the scrotum is a matter of slight importance," and a few lines further on adds, " when *there is no reasonable hope* of the descent into the scrotum being fully and completely accomplished, and when the patient is exposed to the serious inconveniences of hernia," a truss should be applied. And thus the reader who takes Mr. Curling for his guide is left in doubt as to what practice he should adopt in a case of the kind now under consideration. The passage first quoted, in which the case above mentioned is commented on, would induce him to apply a truss to a boy a year old; the last two extracts would incline him to wait until there " was no reasonable hope" of the descent of the testicle occurring, but he will in vain search for any interpretation of the vague phrase " reasonable hope," for any statement of the period when hope ceases, or of the signs by which we may know that it has not fled.

It is not easy to explain satisfactorily why many most eminent and truly excellent surgeons have recommended the practice of allowing a patient to encounter all the perils of an unreduced hernia, with the view of favouring the descent of a testicle. Pott says " I do not know of any particular inconvenience arising from the detention of a testicle within the cavity of the belly," (Surgical Works by Earle, vol. ii, pp. 235-6,) but most inconsistently tells us elsewhere, " while there is a testicle in the groin, *or even within the abdomen*, . . . . the application of a truss would be *highly improper*, for fear of bruising, *or of preventing the descent of the gland*," (Id. Op. vol. ii, p. 117;) that is to say, the rules of surgery and of common sense are to be violated, by permitting a patient to labour under a dangerous disease, for, as we shall presently see, the chance of remedying a condition of things not attended with " any particular inconvenience." Sir A. Cooper, we have seen from pages already quoted, describes an undescended testicle as scarcely, if at all, diminished in size or impaired in organization, and therefore fit for procreation, but of " a testis late in descent, *and protruded by a hernia*" he says, it " is *often lessened in bulk*, but the testis on the other side, with this diminished organ is sufficient for the procreation of children." (Op. cit. p. 46.) We may just observe, that " the testis on the other side" *without* the diminished organ would be quite sufficient for procreation, but our object in quoting this passage is to show that Sir A. Cooper unquestionably intimates that a testicle protruded by a hernia is very likely to be in a worse condition than if it remained quietly within the abdomen, and yet he adds "in those cases in which the testicle has not descended at birth, it often happens that a hernia becomes the means of its descent; and such hernia should remain without a truss being applied, until it has brought down the testis into the scrotum." (p. 46.) Even those who adopted Hunter's views were not quite consistent in their anxiety for the descent of the testicle, for as they attributed its late descent, and still more its complete detention within the abdomen, to an original imperfection of the organ, instead of attributing the supposed imperfection to its residence in the abdomen, it is not easy to see why, on their own hypothesis, they should object to a hernia being retained by a truss, on the grounds that the exit of the testicle would be thereby prevented.

Mr. Curling, as we have seen, questions the propriety of the practice against which we contend, but not, we think we have shown, either with sufficient precision or sufficient decision. Mr. Curling must be aware that he could have fortified his views (which however he omits doing,) by high authority. Not to multiply references we shall merely cite Boyer, (Traité de Malad. Chirurg., t. viii,) and Jobert (de Lamballe), (Traité théor. et pratique des Malad. Chirurg. du Canal Intestin, t. ii, p. 338,) who recommend us to apply a truss to a reducible hernia when the testicle is in the abdomen, with the view of obliterating the hernial opening, without being at all uneasy respecting the testicle, which will perform its functions just as well in the abdomen. If a testicle descended with a rupture, and was adherent to the intestine we believe no surgeon would hesitate to return both into the abdomen if he could effect it, and the liability to such adhesion constitutes another objection to permitting the protrusion of a testicle by a hernia, for if the ring will not permit the return of the gland, the state of the patient is then truly miserable. Another point, to which Mr. Curling does not allude, though his own case above adverted to affords an example of it, is, that after exposing the patient to all the mischiefs of an unreduced hernia, the rupture may fail to bring down the testicle, in which cases, of course, a very serious evil is incurred without obtaining the very questionable advantage for the sake of which that evil has been inflicted. Mr. Curling's proposal to apply a truss on the inguinal ring, when a testicle has not made its appearance at the age of ten or twelve, (years we presume, though this is not specified,) is, so far as we know, original, and we need scarcely say that we think it a very judicious suggestion, but if this precaution is to be adopted, we think it should be taken at a very much earlier age, for we trust we have shown that the great point in those cases is, not to procure the descent of the testicle, but to prevent or remedy rupture.

The only real disadvantage of imperfect descent of the testicle is its liability to injury if in the groin; while, if in the abdomen, disease originating in the testicle may extend throughout that cavity, "there is no shut sack, no distinct tunica vaginalis restricting the limits of inflammation when set up." (p. 84.) This evil, however, cannot for a moment be put in competition with those of an unreduced hernia. Mr. Curling gives two cases as examples of its occurrence. The subject of one of these was a lad aged ten who died with symptoms of acute peritonitis, five days after he had received a kick in the right groin.

"Marks of extensive peritonitis were found throughout the whole of the abdominal cavity, the viscera being coated with lymph, and a turbid serum abundantly effused. In the right iliac fossa, just beneath the peritoneum were seen two small abscesses of recent formation. An atrophied testis was discovered close to the external ring, amongst a mass of cellular tissue, infiltrated with pus and lymph." (p. 85.)

The second case is inconclusive, as the patient was affected with hernia, and recovered, so that its real nature was not ascertained.

In the section on *Diagnosis in cases of imperfect descent of the testis,* our author notices the rare case of a testicle arrested at or just below the external abdominal ring, and complicated with hernia and a collection of fluid in the sac. This case, the most difficult of the kind that can occur, has been particularly described by Dupuytren. The diagnosis can only be determined by carefully considering the distinctive characters of the tumour, and these are not specified in the present work. There is a fluctuating and perhaps a transparent tumour; on gentle pressure the fluctuation disappears, and the tumour diminishes from the return of the serum into the abdomen; the swelling then feels more solid, and further pressure reduces another portion of it with the characteristic sensation felt during the reduction of a hernia; and there then remains a rounded tumour which may be irreducible, or may ascend some way in the inguinal canal, pressure on which causes a peculiar painful sensation. In one case of this kind, (except indeed that no intestine was down,) Dupuytren found the diagnosis extremely difficult; Mr. Curling refers to this case, but does not mention the circumstance which rendered the diagnosis so difficult, and which constitutes the entire interest and peculiarity of the case; though the inguinal canal was open, and there was consequently a communication between the tunica vaginalis and the peritoneum, yet the fluid could not be returned in the abdomen, because the epidydimis though not adherent to, lay against and blocked up the inguinal ring, and on pressure was so firmly impacted in this situation, that no fluid could pass up. In every case of imperfect descent of the testicle, examination of the scrotum of course greatly facilitates the diagnosis. Some cases have been already published in which error resulted from neglect of this examination, and Mr. Curling knows of two instances in which an inflamed testicle situated in the inguinal canal was mistaken for strangulated hernia, in consequence of the absence of the testis from the scrotum having been overlooked. Indeed in every case of doubtful tumour in the vicinity of the genital organs the scrotum should be suspected, for the testicle, as we shall immediately see, occasionally, though very rarely, makes its way into strange situations.

*Descent of the testis into the perineum.* This abnormal situation of the testis occurs very rarely. Mr. Curling mentions the two cases observed by Hunter, and a third communicated to him by Mr. Adams, which are all, he says, with which he is acquainted. How many cases of the kind may be on record we cannot say, but we are only aware of two others, which are briefly noticed by Vidal (de Cassis), (Traité de Patholog. Externe, t. v, p. 655,) and it is worthy of remark that the irregularity affected two brothers, but was not hereditary; the misplaced testicle was in both instances somewhat atrophied.

A still rarer *error loci* of the testicle, if the phrase will be pardoned, is its descent through the femoral canal; Mr. Curling does not mention this unusual occurrence, but two examples of it, at least, are on record. One is mentioned by Vidal, (loc. cit.,) in which the testicle descended directly through the femoral ring, though the patient was also affected with an inguinal rupture on the same side; the other is reported by Eckardt, (Loder's Journ. für die Chirurg. ii Bd. i Stff., p. 187,) in it the testicle first descended through the inguinal canal, and having been returned by the patient into the abdomen, subsequently made its exit through the femoral ring.

The next chapter is on *Atrophy of the testis*, which is treated of under the heads *Arrest of development* and *Wasting.* Our observations on this chapter shall be few, as a very full consideration of the circumstances under which this affection occurs, leads Mr. Curling to conclude, with every previous writer on the subject, "that we have little power by any mode of treatment, to promote the development or prevent the decay of this organ," as its causes "are commonly the result of actions beyond the surgeon's reach or control," (p. 111,) unless indeed when the affection arises from pressure, or impeded circulation, caused for example by an ill-made truss.

It has been said that atrophy of the testicles may be caused by long continued chastity. Mr. Curling at first seems to discredit this notion, as he says that he is not aware of any sufficient authority for the allegation that persons who strictly adhere to their monastic vows become thus affected, and also shows—what has a most important bearing on the question—that deficiency or obliteration of the vas deferens in man and in animals, by no means commonly produces wasting of the gland, (pp. 102-3.) The very next sentence, however, implies that he has seen cases in which disease caused atrophy of the testicles, as it introduces a case, which Mr. Curling says "*is the most remarkable case* I have met with of wasting of the testes, apparently from long-continued inaction," (p. 103. The case is that of a man who had laboured for at least twelve years under disease of the urino-genitory organs, so very severe that he must have been incapacitated for sexual connexion for many years, "both testes were atrophied, being scarcely larger than hazel nuts, but the tubular structure was still apparent. There was a small hydrocele connected with the right testis." (p. 104.) Mr. Curling infers that the protracted sexual incapacity of this individual must have caused "the atrophy of the testes, since the organs were not otherwise diseased." (p. 104.) This case, we think, is a very slender foundation for a doctrine, which is very liable to abuse, and might be perverted into both a temptation to and a plausible excuse for immorality. Independent of the influence of any exhausting chronic disease in diminishing the volume of the testicles, the alterations in the urino-genitory apparatus in this individual, diseased kidneys, contracted bladder, the prostate or multilocular purulent cavity, two urinary fistulæ, the urethra completely impervious and obliterated in one part, stricture in others, abundantly account for the wasting of the testicles, as these organs are very commonly subjected to repeated attacks of inflammation during protracted disease of the urethra, and inflammation is perhaps the most frequent cause of their atrophy. The existence of a hydrocele confirms this view, if indeed confirmation of it were wanting.

Mr. Curling notices inflammation as the most frequent cause of *complete* atrophy of the testicle; but he does not mention, to use Sir A. Cooper's words, "the danger of severe and repeated attacks of inflammation," which do not indeed commonly lead to perfect wasting, but frequently "are followed by a considerable diminution of the virile power, leaving the testicle lessened in its size, and its capacity for secretion." (Op. cit. pt. ii, p. 25.) This is of practical importance, for example in the treatment of a case of disease of urethra, say stricture, in which there is a great disposition to inflammation of the testicle, the utmost care should be taken to prevent its supervention, and when it does occur, to remedy it as promptly and energetically as the condition of the patient will allow.

Several cases have been observed in which the testicles wasted after injury of the head. Mr. Curling refers to those recorded by Larrey, Hennen and Lallemand, and adds a fifth which fell under his own observation. In it, as in the others, the injury had been inflicted at the posterior part of the head. Virility was quite lost, the right testicle was no larger than a horse bean, the left was much diminished in size, and tumours resembling mammæ were developed on each side of the chest. The skull at the occiput seemed somewhat flattened.

*Injuries of the testis* are treated of in the third chapter. The section on *Contusion* leaves nothing to wish for, but *Punctured and incised wounds* are disposed of somewhat too briefly, a rare fault, by the way, with the author.

Formerly wounds of the testis were regarded with too much apprehension, latterly their bad consequences are perhaps rather under-rated. Mr. Curling, we are free to admit, is right in saying that these wounds "are not in *general* followed by severe results," and that "the fact that they commonly do well, should be remembered by the surgeon, that he may not too hastily despair of saving the gland in incised wounds even of a severe character." (p. 114.) But the entire subject is disposed of in nine lines, five of which we have quoted; and there is no mention of the mischief that occasionally though rarely follows these wounds. We suspect that trocars are thrust into testicles oftener than is perhaps thought. Usually all goes well, but inflammation and suppuration sometimes follow, and of this we have seen two instances. Vidal (de Cassis) candidly states, that having a patient at la Charité affected with a tumour of the testicle, the nature of which was very obscure, he punctured it with a very fine trocar. Inflammation set in the next day, and ultimately killed the patient. Mr. Curling does not warn the student, that in incised wounds of the testicle the tubuli seminiferi project through the lips of the wound, and might be, indeed have been, mistaken for a slough or flocculi in the pus. Even J. L. Petit fell into this error, but luckily discovered in time that he was removing the proper substance of the testicle; but others have actually emptied the tunica albuginea.

We pass over the chapter on self-castration, to the fourth, which is devoted to a much more important subject.

*Hydrocele.* Mr. Curling commences this chapter with some observations on the effects of inflammation of the tunica vaginalis on the testicle; he adverts to the opinions of Gendrin, that inflammation of the tunica vaginalis extends to the epidydimis, but not to the body of the testicle, and accounts for this circumstance, by the sub-serous cellular tissue transmitting the inflammation to the epidydimis, while the tunica albuginea arrests its progress to the body of the gland. The alleged fact of the exemption of the body of the testis from any participation in inflammation of the tunica vaginalis, rests, according to Gendrin, on one case. Mr. Curling adopts this view, but does nothing to confirm it, though he had abundant opportunity of satisfying himself on the subject. He refers to a preparation presented to the museum of the College of Surgeons by Sir W. Blizard, which shows the effects of inflammation of the tunica vaginalis caused by the application of caustic for the cure of hydrocele, and he himself examined three testicles shortly after the tunica vaginalis had suffered from acute inflammation, and also nine at a remote period when adhesions only were found, but in every instance he merely describes the condition of the last-mentioned membrane, and says nothing of the condition of the testicle itself. We notice this the more particularly because Sir A. Cooper says "in general, I observe that when there are marks of inflammation *upon the tunics* of the testis—such as, for example, adhesion, the *substance of the gland itself is changed*, the septa are much more apparent than natural, the seminiferous tubes appear to be less in number, are undoubtedly much reduced in size, and many become cords instead of tubes." (Op. cit. pt. ii, p. 23.) These appearances are unquestionably the effect of inflammation propagated to the body of the testicle; and even if this be denied it matters not, we still have the coexistence of adhesion of the tunics and *same prejudicial* influence exerted on the structure of the gland. That such inflammation is not always so propagated is undoubted, but Gendrin and Mr. Curling are wrong in generalizing from a single case, as Sir A. Cooper has *generally* observed the very reverse of the position they maintain. Mr. Curling says that Gendrin's theory "explains the fact that after inflammation of the tunica vaginalis excited by injection, the body of the gland is rarely found to suffer." (p. 121.) That it often, perhaps usually, escapes, and very rarely suffers in any important degree, we admit, but this immunity depends on the moderate amount of inflammation commonly excited. This is not a barren discussion, for in the prevalent rage for deviating from the common track, the operation by injection has not escaped, and some of the old operations for the medical cure of hydrocele, such as incision and the seton, have been proposed as general methods of treatment, chiefly on the grounds that they are less liable to fail than the operation of injection. This cannot be denied; but the knowledge of the fact that severe inflammation of the tunica vaginalis extends to and damages the structure of the testicle, renders it imperative on the surgeon, independently of the propriety of sparing the patient suffering and protracted confinement, to treat hydrocele by the mildest means usually adequate for the purpose.

Mr. Curling first gives the history of *Simple hydrocele of the testis*, for thus he designates what is commonly called hydrocele of the tunica vaginalis. We greatly prefer the latter term, both as being generally received and as conveying a correct notion of the nature of the disease. The symptoms of hydrocele are first described as they ordinarily occur, their occasional variations are then stated, and some of the characters of the affection are noticed in the section on its diagnosis: but, notwithstanding this methodical arrangement, some omissions have struck us. The only exceptions mentioned to a hydrocele being "an oval pyriform tumour," with "a smooth and even surface," are the occurrence of the hour-glass contraction and the irregular shape consequent on adhesion of the testicle to the front of the tunica vaginalis; and there is no notice of the inequalities that occasionally exist on the surface from the tissues of the scrotum yielding unequally to the pressure of the fluid, nor of the deviations from the pyriform shape that sometimes occur, such as the tumour being nearly spherical, or its transverse exceeding its perpendicular diameter. The directions for detecting transparency are by no means adequate to guard the inexperienced against error. We are told that "the mode of making the examination generally adopted is to darken the room and place a lighted candle between, so that the tumour may be interposed between the eye and the light." (p. 138.) But if certain precautions are not adopted, such an examination might very readily mislead. Unless the tumour is firmly grasped behind so as to make it project anteriorly and isolate it from the other side of the scrotum, transparency when it exists may very readily be overlooked; and to avoid falling into the opposite error, it is requisite, especially if the tumour is small, to place the edge of one hand on the front of the tumour to intercept the rays of light, and even then if the hand is placed too obliquely, if the fingers are not firmly applied to the scrotum and to each other, or if either the light or the eye is placed too high, illusion may occur from the light being deflected over the tumour and reaching the eye. Mr. Curling recommends, in cases in which it is difficult to detect transparency, either from the thickness of the parietes of the sac, or from the dark colour of the fluid, the use of "a wooden tube, about three quarters of an inch in diameter, open at both extremities, (or in its absence a roll of writing-paper,) one end being placed against the swelling opposite the light; the surgeon, on looking through the other, can observe the transparency with great advantage. (pp. 138-9.) Such an instrument has been already proposed by Segalas, and we believe others, but has generally been rejected as superfluous, as an error can scarcely be committed, when it is possible to detect transparency, if the rules already adverted to are observed. Mr. Curling does not mention a circumstance observed by Velpeau and Berard, viz. that transparency may disappear without any assignable external cause, and either remain permanently absent, or return. This probably arises from exhalation of blood from the sac.

The *Spontaneous disappearance* of hydrocele, though very common in infants, is very rare in the adult. Mr. Curling refers to four cases of this event, two of which are recorded by Pott, the others by Sir B. Brodie. One of Pott's cases, however, cannot be considered an example of spontaneous cure. The patient received a violent blow on the scrotum, which became "much swollen and very painful," in a word, highly inflamed, so that the cure was caused by inflammation excited by the injury. One of Sir B. Brodie's cases is almost equally open to objection; in it a hydrocele having become painful *was punctured;* inflammation went on for some time, and the hydrocele finally disappeared. It might as well be said that those cases, in which the fluid does not collect again after the operation of tapping or acupuncture, are cases of spontaneous cure. We think it worth observing, that though Sir B. Brodie thus adduces but one unexceptionable case of the spontaneous cure of hydrocele in the adult, a very remarkable case no doubt, as the tumour was so large that the patient, a clergyman, was forced to wear his cassock to conceal it, he yet says, "I every now and then see a case in which a spontaneous cure of hydrocele has occurred," (Lond. Med. Gaz. vol. xiii, p. 90.) We thus see how very cautiously we must take general statements made from memory, and without a reference to particular cases, even when such statements emanate from individuals of the greatest talent, candour, and probity.

We shall not dwell on the treatment of hydrocele by external remedies; Dupuytren occasionally succeeded with blisters, but they always failed with Sir A. Cooper. Mr. Curling says, he twice removed hydrocele by means of external remedies, but both cases are inconclusive. In the first, (pp. 145-6,) the hydrocele *was tapped,* and three days after became tender on pressure; leeches, cold lotions, &c. were employed, and the fluid was absorbed in about three weeks; this is an example of the cure of hydrocele by mild inflammation consequent on tapping. In the second case, the application of tincture of iodine caused the temporary absorption of fluid from a very small hydrocele, but the fluid soon collected again, and the cure was subsequently effected by injection.

Mr. Curling gives very minute directions for performing the operation of tapping a hydrocele, on which we shall make but one observation. He says, "the best place for making the puncture is about the centre of the anterior part of the tumour," (p. 148,) and directs us to "insert the trocar, previously well oiled, perpendicularly into the tumour, with a brisk motion of the right hand," (p. 149;) of course adding that the instrument should be directed upwards when it has entered the sac. We prefer Sir A. Cooper's directions, to "introduce the trocar *two thirds* of the length of the swelling downwards, and *not* horizontally, but with a slight obliquity upwards," (op. cit. p. 180;) and we certainly object to the brisk motion of the right hand, especially if the instrument is held perpendicularly, and if the tumour is small; and we must acknowledge, though many will think it wrong and unnecessary, that we approve of dividing the skin with a lancet previous to passing in the trocar. This may appear to some minute criticism, but we appeal to Mr. Curling, who says, with somewhat of exaggeration no doubt, "simple as the case may appear, the surgeon should omit none of the customary precautions, for more mishaps have occurred in the treatment of hydrocele than in any other operation in surgery." (p. 148.)

*Incision.* This operation, though still performed by some, for example, Rust, Gama, and Jobert, is deservedly rejected by the vast majority of surgeons as a general method of effecting the radical cure of hydrocele. Mr. Curling limits its applicability to cases where "a hydrocele is found to depend on the presence of loose cartilages in the sac," where "it is the only treatment that can be of service." (p. 158.) But injection frequently succeeds notwithstanding the presence of a loose body in the tunica vaginalis, and surely we must admit with Sir A. Cooper, that incision is sometimes indicated "when obscurity hangs over the nature of the case, as to its being connected with hernia, or some enlargement or disease in the testicle," (p. 158;) and we also think that incision is the best operation in some cases where the cyst is multilocular.

*Caustic.* This mode of treatment has, we think, been too completely exploded in consequence of not having been properly applied. To use Mr. Curling's words, "a caustic is applied to the scrotum, so as to destroy the integuments and cause a slough, extending to the tunica vaginalis," (p. 160;) and then follow the just and obvious objections: "It occasions a needless destruction of parts, and is liable to produce a tedious and unhealthy sore: its action cannot be regulated with such exactness as to insure an opening through the tunica vaginalis, so that a fresh application of the caustic, or the introduction of a lancet or trocar was often necessary to complete the process; its operation is slow, and the consequences are unnecessarily severe and painful." (p. 161.)

We fully admit the justice of these objections, but some of them may be removed, and others greatly lessened by a more judicious mode of using the remedy. The caustic should not be applied on the skin; a small incision should be made with the lancet nearly down to the tunica vaginalis, and the edges of the incision being protected with oiled lint, the caustic should be rubbed on the bottom of the wound. We have frequently seen this method, by whom proposed we do not know, adopted by others, and have occasionally practised it ourselves with the most satisfactory results. It is greatly superior, in expedition, certainty, and mildness, to the plan above described, which, objectionable as it is, was yet, Sir A. Cooper tells us, "when well managed, a very powerful operation," though true it is, he adds, "it required great attention to its use, was occasionally severe, and some have known it, in a bad constitution, destroy life." (Op. cit. p. 183.) These last observations, however, every one will admit are applicable to injection or any other operation for the cure of hydrocele; nay, Sir A. Cooper saw two patients die from simple puncture of a hydrocele. We by no means intend to imply that the caustic is preferable to injection in the treatment of hydrocele; even when used as we have described, we admit that it is more painful, more tedious, and more severe: we only mean to say that we are greatly inclined to think it the next best method to injection, and to prefer it when injection has been two or even three times used and failed. It is quite inapplicable however to children, in whom, when discutient lotions fail to disperse the fluid, the seton should be preferred, because a single thread suffices in them; but in the adult when injection fails, a large seton is commonly requisite, and we believe the caustic is usually less severe in its effects than such a seton. We are however well aware of the very extensive experience that is requisite to determine the comparative value of different methods of treatment, and by no means pretend that our own has been sufficient to decide the question.

We must pass rapidly over the remainder of Mr. Curling's chapter on the *radical cure of hydrocele*, the entire of which is, in our opinion, very judicious and well calculated to be a safe and satisfactory guide to the practitioner. Mr. Curling generally uses lime water for injecting a hydrocele, which he says, "though a mild injection usually excites sufficient irritation to cure the disease, and I have rarely had occasion to resort to fluids of a more stimulating nature." (p. 168.) We greatly prefer Mr. Curling's rules for the treatment of the patient after the operation of injection, to those inculcated by Sir A. Cooper. This eminent surgeon told his patient, "If you be in much pain lie down, if you suffer but little take exercise; if you be in much pain, eat very little, and drink only diluents; if you suffer but little, take your dinner, and two or three glasses of wine. Come to me to-morrow." (Op. cit. p. 187.) Mr. Curling's experience, however, does not incline him to rely on the prudence of the patient in regulating his conduct after the operation. "If, as generally happens, symptoms of inflammation arise in the course of a few hours, I usually recommend the use of a suspender to keep the testis supported, and direct the patient to remain in the recumbent position, until the acute symptoms begin to subside. If these precautions be neglected, there is risk of more inflammation being excited than is necessary." (p. 170.) When the inflammation was too low and failed to be sufficiently excited by exercise, handling the scrotum, &c. Sir A. Cooper either tapped the tumour again after a few days, or passed in a seton consisting of a single thread. Mr. Curling does not allude to either of these plans, which, however, often ensure the cure and prevent the necessity of repeating the injection.

M. Baudens has latterly proposed a plan of treating hydrocele, not noticed by Mr. Curling, which we think it well to mention, though we certainly do not anticipate that it will supersede the methods now in use. M. Baudens fits an ordinary acupuncture needle with a canula, which, besides being open at each extremity, is perforated laterally at its centre. This instrument is passed into the hydrocele, brought out about an inch or an inch and a half from the point at which it was passed in, and the needle being withdrawn when the scrotum is thus transfixed, the fluid passes through the central opening into the canula, and thus finds exit. The canula is fixed by twisting a thread round it in a figure of 8, and left in situ for six or eight days. The fluid escapes guttatim, and if obstructed the tube must be cleared. When the fluid passes by the side of the instrument it is withdrawn; the resulting fistula remains open about a week, and when it heals the cure is complete. Such is M. Baudens' account of his method, which of course, like every new proposal, has been most powerful in his hands. Though not strictly connected with the subject immediately under consideration, we may just add, that M. Baudens attaches great importance to this contrivance in the treatment of hydro-sarcocele, as he considers that the smallest quantity of fluid in the tunica vaginalis too small to be removed by any ordinary instrument, materially interferes with the action of medicines on enlarged testicles. (Gazette des Hôpitaux, 1840, p. 531.)

The section on *Congenital hydrocele* is short. As to its diagnosis two circumstances of some importance are omitted; we speak of the disease in the adult. First, that after the fluid has been completely returned into the abdomen, it will usually, though certainly not always, re-accumulate in the scrotum, though the patient remains recumbent. And secondly, that if the patient stands erect when the fluid is in the abdomen, and the finger is kept lightly on the inguinal ring, the tumour will gradually return in the scrotum, though the surgeon has felt nothing pass beneath the finger, and is quite certain that the intestine cannot have descended.

Desault, Dupuytren, and others have injected this form of hydrocele, firm pressure being made on the ring. We have twice seen this operation safely and successfully performed, but we quite agree with Mr. Curling that it should not be practised, on account of the risk of inflammation extending from the tunica vaginalis to the peritoneum. Mr. Curling says, that it actually has excited fatal peritonitis. We were not aware that peritonitis had actually occurred, except when the injection had passed into the abdomen, in consequence of pressure not having been made on the ring. It might be supposed that throwing a wine injection into the abdomen must inevitably excite mortal peritonitis, yet, strange to say, this has not always been the case. Dupuytren (Leçons orales, t. iv, p. 442,) knew of a case in which the patient survived; nay more, in an instance mentioned by Jobert (de Lamballe) (op. cit. t. ii, p. 334,) the symptoms excited were very slight, "Heureusement que le malade en fut quitte pour des coliques, de la sensibilitè au ventre, et quelques symptomes de peritonite."

*Encysted hydrocele of the testis.* This is an accidental serous cyst, such as is often developed in other situations. It occurs most frequently in the epidydimis, and Mr. Curling refers its origin in this situation to the "small serous cysts not larger than a pea, or even smaller, that frequently exist immediately beneath the tunica vaginalis covering the head of the epidydimis." (pp. 185-6.) These accidental cysts sometimes project the tunica vaginalis before them, and became pedunculated. "These pedunculated cysts never acquire a large size," but "when one or more of these cysts, instead of becoming pedunculated, enlarge so as to form an evident tumour in the scrotum, they constitute . . . . *an encysted hydrocele of the epidydimis.*" (p. 187.)

The second variety of the disease is very rare. In it the accidental cyst is situated between the tunica albuginea, and the tunica vaginalis. Mr. Curling has only met with one specimen of this disease, which he discovered accidentally in the dead subject, and only refers to another mentioned by Sir B. Brodie. Sir A. Cooper, though he does not mention this affection, has figured an example of it, plate xi, fig. 4.

Mr. Curling once found in the dead subject, "six or seven small serous cysts, about the size of currants, studding the surface of the loose portion of the tunica vaginalis . . . . If a cyst thus situated, were to increase to any size, it would constitute a swelling which might be appropriately termed an encysted hydrocele of the tunica vaginalis." (p. 190.)

Encysted hydrocele of the testicle is diagnosticated from simple hydrocele, Mr. Curling says, by the different relative position of the testicle and tumour in the two affections, and also by the different nature of their fluid contents, which, according to our author, are always clear and colourless in the former affection. This is not, however, always the case, for Sir B. Brodie has found the serum yellow, "like that in common hydrocele." (Lond. Med. Gaz., vol. xiii, p. 137.) Sir B. Brodie has never known injection, even though repeated, succeed in effecting the cure of this species of hydrocele. Mr. Curling apparently has, for he merely says that injection does not succeed so well as in common hydrocele. As the symptoms are not unfrequently severe during the treatment of this affection with the seton, the palliative treatment should be preferred, unless circumstances, such as the bulk of the tumour, pain, &c., render the former proceeding necessary, and Sir B. Brodie mentions a circumstance, not alluded to by Mr. Curling, which is very favorable to the palliative treatment, that is to say, the slowness with which the fluid for the most part collects, as twelve months or even a longer period may elapse before it returns.

*Diffused hydrocele of the spermatic cord.* Mr. Curling in common with most surgeons does not seem to have met with a case of this affection.

*Encysted hydrocele of the spermatic cord.* In infants this affection originates perhaps always in imperfect obliteration of the process of peritoneum drawn down during the descent of the testicle, and there can be no doubt that it has frequently a similar origin in adults, though it seems equally certain that it occasionally occupies the deep fascia of the cord or common envelope of the cord and testis, derived from the transversalis fascia. Mr. Curling maintains, that, excepting the case of accidental serous cysts, encysted hydrocele of the cord is always situated in the process of peritoneum just mentioned, and in support of this opinion, cites Cloquet's remark, that the remains of this process are frequently met with in subjects of every age, together with some dissections of his own, of the disease itself. We only differ from this view in thinking it too general, and we may just remark that Mr. Curling, we are sure inadvertently, puts it forward as if he had originated it himself, whereas others, for example, Sir A. Cooper, to whom he might, but does not, refer for confirmation of his ideas, have adopted precisely the same opinion.

With respect to the symptoms of this disease Mr. Curling says, the tumour "feels even and tense, and has a manifest fluctuation." (p. 207.) But it has been generally observed that fluctuation is not a manifest character of this tumour, being on the contrary very obscure and often scarcely or not at all to be detected ; the reason of this being that the tumour is usually "tense," as Mr. Curling himself correctly states, or, to use Sir A. Cooper's words, "extremely firm to the feel." (Op. cit. p. 196.)

As to the *Treatment*, Mr. Curling observes that injection "is not much in favour with practical surgeons." Velpeau, however, prefers it, unless several cysts exist, or unless the tumour extends into the inguinal canal ; but we quite agree with the author that the seton is the better method of treatment.

A section is devoted to the complications of hydrocele of the tunica vaginalis with encysted hydrocele of the testis, encysted and diffused hydrocele of the spermatic cord, and of hydrocele whether of the tunica vaginalis, or of the spermatic cord with hernia. This section is very well executed, but calls for no particular remark.

*Hydrocele of the hernial sac*, is a term which has been differently used by different writers. Some, as Scarpa, extend the term to any considerable accumulation of serum in a hernial sac, even when occupied by intestine. Mr. Curling says, "the term should, I think, be restricted to cases of a chronic collection of fluid in the sac of an old hernia, in which the communication has been permanently obliterated by adhesion at the neck, either of the sides of the sac, or of a portion of omentum or intestine." (pp. 229-30.)

Mr. Curling has, we believe, been rather generally anticipated in this opinion, at least since Boyer insisted on the distinction, that the coexistence of fluid with a hernia was a sheer accident grafted on the chief disease, and that the term in question should be limited to cases in which the sac has been long abandoned by the hernia, its neck become obliterated, and fluid accumulated in its interior, constituting what he calls "hydrocele du sac hernienne sans hernie." Mr. Curling proposes the name *spurious hydrocele of the hernial sac*, for "all cases of a hernial descent coupled with serous effusion," (p. 230;) but we do not see the necessity to designate an accompaniment of hernia, so very common that it constitutes a necessary part of the history of that complaint; and in fact Mr. Curling himself observes, "nothing is more common than the presence of fluid in the sac of a strangulated hernia." (p. 230.)

Respecting the diagnosis of this complaint, Mr. Curling says,

"I conceive that some little difficulty might be experienced in diagnosing a *small* hydrocele of the hernial sac from an encysted hydrocele of the cord high up, . . . . a hydrocele of the hernial sac occurs somewhat late in life, is usually of some considerable size, and its fluid contents are of an amber or dark colour, whilst an encysted hydrocele of the cord generally appears before puberty, is rather small in size, and contains fluid which is generally colourless, and nearly free from albumen. Attention therefore to these distinguishing marks, and to the history of the case, would leave but little room for doubt." (p. 227.)

Now we believe, with Boyer, that there is not some little difficulty, but great difficulty, in distinguishing by any external signs hydrocele of the hernial sac from encysted hydrocele of the cord extending to the inguinal ring, in fact that the diagnosis can *only* be effected by a reference to the history of the case: *all* the other "distinguishing marks" above enumerated are illusory. Hydrocele of the cord occurs late in life; hydrocele of the hernial sac may be small in size, and its fluid contents may be colourless.

We pass by the section on *Hydrocele in the female*, and have only to observe respecting the chapter on hæmatocele, that the first section is headed *Hematocele of the testis*, and yet the section commences with the words "in hematocele of the tunica vaginalis:" why then does not the author in the first instance adopt the latter more accurate and generally received term? We mention this the rather, because Richter admitted as a variety of hæmatocele, extravasation of blood beneath the tunica albuginea, and though this has been rejected as a fanciful or at least an unnecessary refinement, or rather because it has been thus rejected, the term should not be applied to another and quite different affection.

The account of the pathology, symptoms, diagnosis, and treatment of the several forms of hematocele is not merely satisfactory, but excellent.

Few diseases occur more frequently than inflammation of the testicle, or *Orchitis*, which forms the subject of Mr. Curling's sixth chapter. We cannot go through this chapter in detail, but will confine ourselves to a few of the more important points of the history of the disease, and take occasion at the same time, for the sake of those who settle every point by an appeal to *authority*, to examine how far the *authorities* are agreed respecting the very nature, mode of origin, and some of the most obvious phenomena of this very common disease.

Mr. Curling observes that he is "unacquainted with any authentic account of the alteration in structure from inflammation originating in the body of the gland;" (p. 254 ;) he has, however, "twice been able to inspect a testis affected with acute secondary orchitis;" and from the examinations together with the account of two cases dissected by M. Gaussail, (Archiv. gén. de Méd., t. xxvii, p. 210,) gives the following description of the pathological appearance, which we insert in full, as M. Gaussail's dissections—the only ones, so far as we know, hitherto recorded—having been published in a voluminous foreign journal, are not generally accessible.

"The tunica vaginalis is more or less distended with lymph, or albuminous matter infiltrated with reddish serum, which form loose adhesions between the opposed surfaces of the membranes: these adhesions are so slight as easily to admit of being broken down with the finger. The membrane is injected with a multitude of minute red vessels, which ramify in various directions, and form a compact network. At a later period red vessels may be traced proceeding from the free surface of the tunica vaginalis to the false membranes forming the adhesions. The volume of the testis appears very little if at all increased, the great bulk of the tumour being occasioned by the effusion into the serous sac. When cut into the gland appears somewhat darker than natural, from a congested state of its vessels. The epidydimis, particularly the lower part, is enlarged to twice, and sometimes thrice its natural size, and feels thick, firm, and indurated. This enlargement is produced by the effusion of a brownish deposit in the cellular tissue between the convolutions of the duct. The coats of the vas deferens are thickened, and the vessels ramifying near them injected sometimes along the whole extent of the duct. Albuminous deposit is found in the cellular tissue around the tortuous part of the vas deferens and tail of the epidydimis, which frequently forms the bulk of the swelling observed in these cases. Owing to the epidydimis being the part chiefly and most constantly affected in consecutive orchitis, some of the modern French writers have denominated the disease epididymitis." (pp. 254-5.) Subsequently Mr. Curling adds, "I do not mean to imply, however, that the glandular structure of the testis never suffers in consecutive orchitis, for I believe that it does so in some instances, but according to my observations, and I have paid some attention to the subject, it very commonly escapes, the inflammation not extending further than to the epidydimis." (p. 256.)

The exact nature and situation of the swelling in acute secondary orchitis has within these few years given rise to a great deal of controversy. It was always known that much of the tumefaction was formed not only by the swollen epidydimis, but by effusion into the cavity of the tunica vaginalis; but it was also taken for granted that a considerable, perhaps the greater, part of the tumour consisted of the body of the testis. Thus Sir A. Cooper says, "the testicle swells and soon increases to two or three times its natural bulk," (op. cit. p. 9,) and "although the testicle is *very much swollen*, it still retains its original form, being rounded on its fore part, but somewhat flatter on its sides, and it feels excessively hard," (p. 10,) and the peculiar kind of pain experienced in this affection was generally accounted for by the compression exerted by the tunica albuginea on the swollen glandular structure of the testicle. This doctrine we believe was never questioned until Rochoux in 1833, (Achiv. gén. de Méd., t. xxxii, p. 51,) maintained that the body of the testicle was not affected in gonorrheal orchitis, that the disease consisted in inflammation of the tunica vaginalis, and should therefore be called *vaginalitis*, and that effusion into this serous sac formed the great bulk of the swelling to which the epidydimis contributed in but a trifling degree. M. Marc Moreau, (Journ. Hebdom. 1834, t. ii, p. 218,) also contended that the body of the gland is unaffected except in rare cases, but considered the affection as an acute inflammation of the vas deferens and epidydimis which implicated the tunica vaginalis; and this is the view adopted by Ricord, who proposes to term the malady *epidydimitis.* Others, however, still adhere to the old opinion : for example, Velpeau has satisfied himself, from the examination of above 100 cases, that the inflammation extends to the body of the testicle as well as to the tunica vaginalis, and that though the effusion into the tunica vaginalis forms from a third to one half the bulk of the swelling, in about half the cases it is frequently very slight, and occasionally quite absent. (Dict. de Méd., 2e ed., t. xv, p. 445.)

Mr. Curling takes no notice of this discussion, but, as we have seen, adopts the opinion of M. Marc Moreau. For our own part we too have paid some attention to this subject, and are persuaded that the body of the testicle participates in the inflammation when the disease is fully developed. This question we think is susceptible of being determined by examination of the disease during life, but if dissections must be appealed to, they also establish that the glandular structure of the testicle is affected. The dissections of the disease in its early stage are too few to admit of any general conclusion being grounded on them, and they are contradicted by the numerous examinations that have been made of those organs at a remote period after the subsidence of the inflammation. If this were merely a question of the opinions entertained by different individuals it would be little worth dwelling on, but we conceive that it is otherwise important; for if the body of the testicle so rarely participates in the disease, its structure can be very rarely affected by it; but we believe that the direct contrary is the case.

When speaking of inflammation of the tunica vaginalis, we showed that Sir A. Cooper generally found marks of inflammation of that membrane, such as adhesion, to coexist with an alteration in the structure of the gland. Let us now see what Mr. Curling says respecting the condition of the organ after it has been inflamed:

"In many instances, after acute orchitis has subsided, the testis is restored to its natural condition; in other cases, permanent changes of a serious nature are the consequence. I have observed in testes that have been affected with inflammation some time before, that the septa appear to be more distinct, and to enter more largely into the composition of the gland than is natural; that the small seminal tubes are less numerous and apparent; and that a great part of the organ is converted into a dense white fibrous tissue without the presence of tubuli." (p. 258.)

This passage, which, by a coincidence no doubt fortuitous, is nearly identical with one which we formerly quoted from Sir A. Cooper, is followed by an account of the appearances observed by Sir B. Brodie, in a testicle which had been affected twenty years previously with gonorrheal orchitis, appearances identical in kind, though greater in degree, with those described by Sir A. Cooper and Mr. Curling. But our author goes on to advert to differences between the remote effects of gonorrheal orchitis, and that which originates in the body of the gland.

"Consecutive orchitis" he says, "*seldom* subsides without leaving behind distinct traces of its existence, which never disappear during the remainder of the patient's life." [These traces are chiefly enlargement of the epidydimis with thickening and dilatation of its duct.] "These changes are *rarely found* without the presence of *old adhesions* obliterating partially or completely the sac of the tunica vaginalis. . . . . The alterations noticed in the body of the testis have been observed, in some instances, coexisting with those in the epidydimis, but in by far the majority of cases, the glandular structure is unimpaired. In only two cases in which the epidydimis was thus diseased, have I remarked *a decidedly atrophied condition* of the organ. The absence of pressure, owing to the unresisting nature of the membrane investing the epidydimis, appears to prevent the obliteration of the duct of which it is composed, and thus accounts for atrophy occurring much more rarely after consecutive orchitis than after inflammation originating in the body of the gland, where the delicate seminal tubes are inclosed in a firm unyielding tunica albuginea." (pp. 259-60.)

On this passage we have to remark that undoubtedly *atrophy* of the testis very rarely follows gonorrheal inflammation of that gland, but we may observe that very serious alterations of its structure are often caused by inflammation consecutive on other diseases of the urethra, simply because those other diseases being chronic or permanent, the organ is liable to repeated attacks of inflammation, each of which inflicts a cumulative alteration on its structure. To return, however, to the consequences of gonorrheal inflammation of the testicle: no one pretends that it produces "a decidedly atrophied condition of that organ," save in very exceptional cases; but the question is, does it usually, or very often, alter the structure of the body of the gland in a minor but yet perceptible degree? And this question cannot be disposed of by the general statement that "in the majority of cases, the glandular structure is unimpaired," especially when Mr. Curling's own statements combined with those of Sir A. Cooper, go to establish the opposite conclusion. Mr. Curling distinctly states that gonorrheal orchitis *seldom subsides* without leaving certain changes in the epidydimis, which changes are *rarely found* without *adhesions* of the tunica vaginalis; but we have the testimony of Sir A. Cooper, that such adhesions are generally accompanied with diminution of the size of the testicle, and alterations of its substance. It will readily be admitted that such appearances are the remote effects of inflammation, and we are therefore justified in concluding that the body of the testicle participates in gonorrheal orchitis, at all events much more frequently than our author with several modern writers admit. It is right to observe that we attach more importance to Sir A. Cooper's evidence on this point, simply because it is positive or affirmative, while that of Mr. Curling is negative; and negative evidence is worth nothing unless founded on minute and carefully-described examinations, and above all unless we know, if not the exact number, at least something near the number of observations on which it is founded.

The origin of gonorrheal orchitis has been referred to metastasis, sympathy, and continuous extension of the inflammation from the urethra to the testicle. This is a point on which we shall find the authorities sadly at variance, and it will be worth while to state a few of the discordant opinions of men of the most deserved eminence and greatest practical reputation.

Dupuytren admits the three modes of origin above enumerated: that by metastasis he considers the most frequent, and says the sudden cessation of the discharge from the urethra, is its pathognomic sign. In the sympathetic variety the discharge, he says, is little if at all modified, the inflammation usually affects the tunica vaginalis, and the scrotum is greatly swollen. Propagation of the inflammation by continuity of surface occurs, he says, but rarely, and is characterized by an inflammation of the testicle, "in which the tumour, very hard, very painful, moveable and always small, presents a prominence formed by the swollen epidydimis. This last phenomenon seems to us a character peculiar to gonorrheal orchitis produced by continuity of tissue, for we have *never* met with it in those caused by external violence, or in cases where the inflammation was suddenly transported from the urethra to the testicle." (Leçons Orales, t. iv, p. 221.) Sir B. Brodie and Ricord admit only the first and third forms of the disease; Ricord says nothing as to their comparative frequency, but Sir B. Brodie considers the metastatic variety the usual one, and that by extension of inflammation as very rare, (Lond. Med. Gaz., vol. xiii, p. 218.) Cullerier, writing in 1830, says, the theory of metastasis is abandoned, and that the inflammation is always propagated by continuity of tissue; (Dict. de Méd. et Chirurg. Prat., t. iv, p. 157;) but in 1834, he informs us that metastasis occurs in nine cases out of ten. (Dict. de Med. ou Repért. gén. etc., 2e ed., t. xii, p. 267.) Sir A. Cooper says indeed that the disease "arises from sympathy with the urethra," but distinctly describes it as solely caused by continuous extension of the inflammation, and Velpeau also, not to mention others, ascribes it to this last cause exclusively. We may just add that Dupuytren, Sir A. Cooper, and Sir B. Brodie, say the discharge from the urethra either usually or frequently ceases, as the inflammation of the testicle supervenes, while Cullerier and Ricord maintain that it never entirely disappears.

Mr. Curling differs from all the authors whose opinions we have cited, that is to say, so far as he gives a decided opinion, for he recognizes only the second and third forms of the affection, or that caused by sympathy, and that depending on continuous propagation of the inflammation. As to metastasis he says, "it is extremely questionable whether anything of the kind ever takes place." (p. 264.) The most frequent cases are those "in which inflammatory action may be gradually traced creeping along the vas deferens to the epidydimis." (p. 264.) "The sympathetic form of gonorrheal orchitis," is that "in which the testis is attacked, apparently without any previous affection of the vas deferens." (p. 267.) These last, Mr. Curling says, are the cases that have been attributed to metastasis, a doctrine "which we cannot readily admit," when we consider how readily and rapidly inflammation may be propagated along a continuous membranous surface without remaining fixed at any part of it long enough to produce any signs of disease, how rarely the symptoms of gonorrhea entirely subside as the orchitis becomes developed, and how seldom the sudden arrest of the discharge by medicine or by injections, is followed by orchitis; and finally Mr. Curling argues that the marked amelioration of the symptoms of gonorrhea that so often occurs cannot be considered as a proof of metastasis, as inflammation of one part is often relieved by the occurrence of inflammation in a neighbouring part, and he once saw a case in which orchitis caused by a *blow* on the testicle was followed by the subsidence of gonorrhea, with which the patient happened to be affected.

The length to which this article has already extended precludes us from discussing Mr. Curling's arguments, and we can only say that we consider the occurrence of metastatic gonorrheal orchitis as firmly established as any example of metastasis of inflammation can be. Its existence no doubt can only be inferred from the observation of symptoms, but the facts expressed by those symptoms cannot be explained away by a reference to circumstances, *not one of which is strictly analogous* to the fact with which it is compared and is intended to illustrate.

We have mentioned the different opinions of eminent practitioners as to the connexion between, or the symptoms of orchitis and those of gonorrhea. On this point Mr. Curling gives an abstract of the researches of M. Gaussail, (Archiv. gén. de Méd., t. xxvii, p. 188,) M. d'Espine, (Mém. de la Soc. Méd. d'Emulation, t. i, p. 426,) and M. Aubry, (Archiv. gén. de Méd. 1841, Mai, p. 23,) from which it appears that in 125 of 154 cases observed by MM. Gaussail and Aubry, the discharge diminished at the commencement of the attack, and 1 in 6 out of 29 cases observed by M. d'Espine, the discharge underwent no change, in 22 was either increased, diminished, or suppressed, and in 3 cases only did it after having been suppressed at the commencement of the affection, reappear as the acute symptoms of orchitis subsided.

There are some remarkable phenomena of this disease which Mr. Curling notices: how rarely for example both testicles are simultaneously affected, and its liability to pass from one testicle to the other. It has been almost generally believed that the left testicle is most frequently affected, but the observations of MM. Gaussail and Aubry, and of Mr. Curling himself, would lead to the opposite conclusion, as in 138 cases they found the right testicle affected in 78, the left in 49, and both in 11, but in most of the last cases, the inflammation had passed from one gland to the other.

Mr. Curling "does not recollect having met in medical works with any notice of acute inflammation attacking the testes of young infants. I have seen, however, a few cases of orchitis at this early period." (p. 272.) An account is given of two cases, one in an infant aged five months, the other in a boy two years old. They both soon yielded to suitable antiphlogistic treatment.

The directions for the treatment are exceedingly judicious; but there is one point that we must notice. Mr. Curling says the value of mercury in the treatment of consecutive orchitis, "scarcely appears to be fully appreciated by the profession." (p. 280.) We cannot agree in this, for certainly our observation would have led us to say it is often given in cases when it could be dispensed with. We utterly disagree with Mr. Curling "that mercury is indicated because of the inflammation of the tunica vaginalis, as reason would lead us to expect from its remarkable efficacy in inflammation of other serous membranes." (p. 280.) If this were all it would be quite unjustifiable to salivate the patient. We agree, however, with the author that in acute cases, mercury given until the gums are slightly affected, often materially abridges the duration of the disease, and "what is of some importance"—we would say of great importance—"is succeeded by much less induration and thickening of the epidydimis." (p. 280.) To this we would add, as by far the most important point, that the use of mercury tends to prevent the alterations of the body of the gland to which, we think we have shown, it is liable.

At the close of this chapter Mr. Curling notices Mr. Ramsden's views as to the connexion between disease of the urethra and chronic disease of the testicle. That Mr. Ramsden's views were monstrously exaggerated is universally admitted, and the exaggeration has perhaps caused them to be too completely neglected; Mr. Curling we think appreciates them very fairly, but as they exclusively related to chronic disease of the testicle dependent on what Mr. Ramsden terms "a latent principle of irritation within the urethra," they would, we think, have been more appropriately noticed in the next chapter on chronic orchitis.

The disease termed "chronic orchitis" by Mr. Curling, is that called "chronic inflammation" and "simple chronic disease of the testicle" by Sir A. Cooper. Sir B. Brodie names it "chronic or *tubercular* inflammation of the testicle, because it ends in the formation of a yellow tubercle in the testicle," (op. cit., p. 220;) and Cruveilhier terms it "tubercular sarcocele," and "tubercular infiltration of the epidydimis."

Mr. Curling objects to the yellow deposit which characterizes this disease being termed tubercle, "as it differs from tubercular deposit, which is also developed in the testis, and is liable to lead to error." (p. 295.) Sir A. Cooper describes this deposit as "yellow fibrin, or coagulable lymph," and Mr. Curling says "it appears to be coagulable lymph" condensed by the tunica albuginea resisting distention. There can, we think, be no doubt that the name chronic inflammation or chronic orchitis should be preferred for this disease, and the term "tubercular" confined to scrofulous diseases of the organ.

Sir A. Cooper and Cruveilhier state that the peculiar yellow deposit seen in this affection occupies the cellular membrane between the tubuli seminiferi. Sir B. Brodie is of opinion that it is secreted from the inner surface of the tubuli testis, as he discovered the yellow substance in the vas deferens and canal of the epidydimis, which are continuous with the tubuli. Mr. Curling gives a minute account of the examination of a testicle, (pp. 291-4,) in what seemed to be an early stage of the affection, which he thinks enables him to confirm satisfactorily Sir B. Brodie's opinion.

Mr. Curling proposes for the fungous growth, or granular swelling which sometimes protrudes in the advanced stage of this affection, the name of "*hernia testis*, being formed in a manner very analogous to that of *hernia cerebri*, in which the substances of the brain is protruded through an ulcerated opening in the dura mater." (p. 296.) Both the suggestion and illustration are borrowed, but without acknowledgment, from Sir A. Cooper, who says, "the principle upon which it (the granular swelling) is founded, is the same as the granular swelling of the brain succeeding a wound of that organ, compressed, when swollen, by the bones of the skull and by the dura mater," (op. cit. p. 36,) and he calls it a "hernial granulatory protrusion." (p. 38.) Nevertheless we think the name improper, or at least unnecessary.

The account of the symptoms, diagnosis, and treatment of this disease is excellent. We have only to observe that though Mr. Curling questions the propriety of the practice recommended by Mr. Lawrence, and occasionally adopted by Sir A. Cooper, of slicing off the *granular swelling or fungus* of the testis, he scarcely does so as decidedly as we could wish. Sir A. Cooper says the granular swelling "is formed of common granulations only," (op. cit. p. 36,) and we therefore can understand why he was induced to make "an elliptical incision in the skin around the projecting granulations," and then carry the knife "under the whole of the swelling, and close to the tunica albuginea, by which the part is excised, leaving the epidydimis and *testicle uninjured.*" (Op. cit. p. 45.) With respect to this practice Mr. Curling says, "I must confess that the operation of excising it scarcely seems to me to be a very scientific mode of proceeding, seeing that the fungus partly consists of tubuli seminiferi, and in some instances includes nearly the whole of the glandular part of the testis, so that its removal becomes an operation which in effect is but little less than that of castration." (p. 317.) Now this is a case for decidedly stating a positive rule of practice, that the fungus is by no means to be interfered with, and Sir A. Cooper should be cited not hesitatingly to question his authority, but to prevent his authority leading the practitioner astray, by stating that in this particular he was unquestionably wrong, because he thought the fungus consisted "of common granulations only." We may just add that Sir B. Brodie, without any reserve, says "the fungus should not be removed, as by doing so we slice away the tubuli testis." (Loc. cit. p. 222.)

The pathological characters and symptoms of "syphilitic orchitis" are far from being satisfactorily determined.

Mr. Curling has had no opportunity of dissecting a venereal testicle, but he refers to Sir B. Brodie, who examined one and found the morbid appearances to correspond with those observed in chronic inflammation. Cruveilhier, we may observe, also says that exactly the same morbid alterations occur in the testicle in syphilis, chronic inflammations after contusions, etc. Mr. Cusack has published the results of the dissection of about twelve syphilitic testicles, which, though very far from being detailed with sufficient precision and clearness, we subjoin, and which to a certain extent confirm the observations of Sir B. Brodie and Cruveilhier. The testicles examined exemplified, we are told, the progress of the disease "from a small circumscribed tubercle in an otherwise sound testis, to the contracted, indurated, and completely disorganized gland. The structure of the tubercle is rather soft, but harder than common scrofulous tubercle, and surrounded by a thickened layer resembling a cyst, the product of inflammatory action. In one preparation the tubercle is in the lower part of the testis, which is otherwise so sound that the epidydimis admitted of injection by mercury." (Dublin Journ. of Med. Science, vol. viii, p. 304.)

A great number of writers have attempted to give a description of the local character of the venereal testicle, but so far as we can recollect they almost all conclude by saying that a diagnosis can only be effected by the co-existence of other venereal symptoms, or by reference to the history of the disease. Dupuytren says the tumour is elongated, cylindrical, and painless when handled, but that its *pathognomic* character is the swelling leaving one testicle after affecting it for perhaps months, and suddenly attacking the opposite one. (Leçons Orales, t. i, p. 108.) Ricord says the tumour is hard, pear-shaped, sometimes uneven and relatively heavy, that the epidydimis or cord may be implicated, but that the body of the gland is always affected. Without further specifying individual opinions, it is enough to say that many, we believe most, writers describe the swelling as commencing in the body of the testicle, and gradually involving it and the epidydimis in one common tumour so that these two parts cannot be distinguished ; this tumour is said to be smooth and uniform on the surface, of a fleshy or firm feel, but not extremely hard, not very weighty in proportion to its bulk, and very little prone to suppuration ; but the symptoms are admitted to be so variable, that the diagnosis, as already said, can only be established by the coexistence or antecedence of venereal symptoms.

Mr. Curling does not attempt any particular description of the local characters of this disease of the testicle, which he says " only differs from those of chronic inflammation of the gland, in the testis becoming occasionally more tumid and painful during the evening exacerbations, or in the occurrence in some cases of nocturnal lumbar pains." (pp. 230-1.) We freely admit that Mr. Curling may be right in not attaching any importance to the local symptoms above enumerated ; yet as we know from personal observation that they do occur, we are inclined to think that it would have been better to have mentioned them. The commencement of the affection in the body of the gland, and its termination in suppuration are, we should mention, specified. Mr. Curling says " the enlargement takes place slowly :" undoubtedly it usually does, but not unfrequently the affection comes on rather suddenly.

" Tubercular disease," properly so called, more frequently affects the epidydimis than the body of the gland. The precise anatomical situation of the morbid deposition is not exactly determined. Mr. Curling has examined many testicles affected with strumous disease, and has " certainly seen this (the tubercular) deposit in the vas deferens near the testis, and in the interior ducts forming the epidydimis." (p. 325.) He, however, thinks that tubercular matter is " deposited within as well as between the tubuli." (p. 326.)

Mr. Curling's account of the symptoms of scrofulous disease of the testis is in our judgment the very best we have met with ; superior, we think in minute accuracy and fidelity to that of Sir B. Brodie (loc. cit.) which is the next best we are acquainted with. On this account, and also because we have not yet given any connected examples of Mr. Curling's manner of describing disease, we insert the following quotation :

" The disease commences insidiously, and is insidious in its progress. The patient's attention is usually first attracted by a slight uneasiness in some part of the gland, generally the epidydimis, which on examination is found to be somewhat enlarged, prominent, and hardened. Sometimes the whole organ feels slightly enlarged and indurated, though it more frequently forms a tumour with an unequal and irregular surface. The state of the testis, however, is often marked by small local effusions of fluid in the tunica vaginalis, the surfaces of this membrane being partially adherent. Very little pain is experienced in the part, and there is but slight tenderness on pressure. After the disease has lasted for some time, many months or even a year and more, making little progress, and often remaining stationary, one of the prominences begins to increase so as to be observed externally, and to feel painful and tender ; the skin over it becomes adherent, changes to a livid hue, ulcerates and bursts, giving vent to a soft caseous matter mixed with pus. This is followed by the formation of a fistulous sinus, which discharges a scanty thin serous pus, mixed with particles of tubular matter and often with semen, particularly after venereal excitement. Similar changes may take place in other parts of the testis, occasioning two or more sinuses leading to the interior of the gland. These sinuses sometimes communicate, and they may continue open and discharging for a great length of time. After the deposit has all come away, if the original disease be arrested, and no more tubercular matter formed, reparative changes sometimes take place; the discharge ceases, the fistulæ close up, leaving the organ more or less diminished in size or entirely wasted, according to the extent to which it had been disorganized by the tubercular deposit. The bursting of the abscess and escape of the tubercular matter are sometimes followed by a hernial protrusion of the testis, as after chronic inflammation of the gland." (pp. 328-9.)

The directions for the treatment are judicious and practical. Mr. Curling says nothing of the use of the liquor potassæ which Sir B. Brodie has found more useful than anything. We mention this the rather that we have derived singular benefit from the use of medicine not only in this affection, but generally in cases of external tubercular disease; but it must be given in large doses, gradually augmented to sixty or eighty drops three times a day if possible. We feel satisfied (though well aware how difficult it is to fairly estimate the therapeutic influence of remedies in chronic diseases) that this medicine, given as freely as the case will allow, in combination with the iodide of potassium, effects greatly more than the latter medicine will accomplish by itself.

Mr. Curling has never seen a case of scirrhus of the testis, and merely gives an abstract of Sir A. Cooper's account of this disease, and we therefore pass by this chapter. The section on *Encephaloid cancer of the testis* is about the best account of the disease which we have met with, and is followed by a short account of that rare affection, "carcinoma of the tunica vaginalis." The still rarer diseases *Colloid cancer and melanosis of the testis* are despatched in a few lines; but we do not know that more could have been properly said about them. With respect to the disease described by Sir A. Cooper, under the name of *Hydatid or encysted disease of the testicle*, Mr. Curling observes that the phrase *hydatid* is here improperly employed, and prefers the term *Cystic sarcoma or cystic disease of the testis.* As his account of the affection is but an abstract of Sir A. Cooper's, we need not dwell on it. We also pass the very brief and unimportant (yet necessary) sections on *Fibrous transformation of the testis*, *Ossific deposits in the testis*, *Loose bodies in the tunica vaginalis*, *Spermatocele*, *Fœtal remains in the testis*, and *Entozoa in the testis.*

Mr. Curling considers the nervous affections of the testis under the heads *Irritable testis* and *neuralgia of the testis*, both of which Sir A. Cooper evidently comprised under the former appellation. The account of the symptoms of irritable testicle closely corresponds with that given by Sir A. Cooper; in it "there is merely morbid sensibility; pain seldom being experienced whilst the patient remains at rest, and the gland and spermatic cord are supported and entirely free from pressure or rough contact with the dress," (p. 380;) but in neuralgia "the pain is sudden, severe, and remittent, and occurs in paroxysms of variable duration, generally at irregular but occasionally at regular intervals. The pain is sometimes of an acute, darting, or lancinating description, at other times of a dragging or pricking nature, and it is commonly attended with forcible retraction of the testis to the groin by the spasmodic action of the cremaster muscle, and occasionally with nausea and vomiting," (pp. 380-1.) Sometimes the testicle can be freely handled, but morbid sensibility may coexist, and then the slightest touch may bring on a paroxysm. Usually no alteration is perceptible in the gland, but in an aggravated or old case it may become swollen, tender, or even slightly inflamed. Neuralgia is almost always confined to one side; morbid sensibility often implicates both. Sir A. Cooper considered irritable testis generally as "of the nature of tic douloureux," (Op. cit. p 64.) Mr. Curling refers the "neuralgia" alone to this head, and says the "irritable testis is in general intimately connected with the state of the genital functions, and is frequently dependent on abuses of them," (p. 376.) As causes, he mentions onanism, involuntary seminal emissions, cessation from free indulgence in sexual intercourse, sexual excitement without indulgence, and occasionally orchitis. "Though troublesome, it generally disappears either spontaneously or under treatment after a longer or shorter duration," (p. 377.) In the treatment Mr. Curling recommends tonics, cold bathing, the douche, belladonna-plaster, and, when there is morbid sensibility of the prostatic portion of the urethra, the application of caustic to that part of the canal by means of Lallemand's porte-caustic, by which he cured three cases. He properly deprecates castration in any case of this disease. Little is known of the causes of *neuralgia* of the testicle. Mr. Curling mentions as occasional causes, varicocele, derangement, of the digestive organs, gout, and orchitis. As to the treatment, it must be directed to the cause where it can be detected, and if it cannot, must be empirical. Quinine, arsenic, iron, oil of turpentine, and narcotics, both internally and locally, may succeed—but more frequently fail. In aggravated and obstinate cases castration has frequently been performed, but Mr. Curling remarks, "Operations, however, for the cure of neuralgia are in general very precarious and unsatisfactory, and the more extended our experience, the less encouragement we find to repeat them. ...... We find that in several of the cases in which the operation has been resorted to, no benefit has resulted from it." (p. 385.) The results of the three cases operated on by Sir A. Cooper were, however, satisfactory. Mr. Curling examines these three cases "in order that the practice of so eminent a surgeon may not be made to countenance the indiscriminate performance of an operation, the general results of which are less satisfactory than might be inferred from these examples." (p. 389.) In all of them Mr. Curling thinks the operation succeeded because the disease was of local origin, varicocele having been present in one, and in the two others the affection having apparently been induced by orchitis. We would account for the success of the operation in the second and third of Sir A. Cooper's cases, from the fact that these cases were examples not of neuralgia, but of irritable testis.

In a chapter on *Sympathetic and functional disorders of the testis*, the subjects of *Impotency* and *Involuntary seminal emissions* are treated of. We deem it unnecessary to give an analysis of the observations on the former affections, but we venture to say that they contain everything that is known respecting it, and will prove very valuable and satisfactory to the practitioner who may need a reference on the subject. The latter has been so amply discussed in the Number of this Journal for April 1843, that it is wholly unnecessary to recur to it. We must find room, however, for the following observations on the occasional connexion between a morbid condition of the prostatic portion of the urethra, and certain debaucheries too often attributed to moral depravity exclusively:

"One would be loth to offer any apology for the vicious habits and indulgences to which it is well known old men are occasionally addicted .......... I cannot but think, however, that in many instances these cases are not undeserving of professional sympathy, and that the erotic longings, which sometimes continue to distress the aged long after the period at which in the course of nature they should have ceased, depend as much on physical infirmity as mental depravity, the former inciting and producing the morbid desires. If these propensities were regarded and treated as symptoms of disease, (and that they frequently occur in connexion with affections of the urinary passages is well known to practical surgeons,) I believe they would often subside, and the distressing results to which they lead would be altogether avoided. (pp. 414-5, Note.)

The chapter on *Castration* requires no comment. We shall make a few observations on that on *Varicocele.*

Within the last few years many surgeons have exerted their ingenuity in devising methods, more or less novel, for the treatment of varicocele; and we think that it will no doubt continue very fashionable to operate on this disease until a few fatal cases of phlebitis gain publicity, and then interference will be deprecated—to be renewed, however, again after the lapse of a suitable period. Mr. Curling, we must observe, is not at all chargeable with encouraging unnecessary meddling with this affection; on the contrary, he expressly limits operation to "the few instances in which palliative means fail to afford relief and arrest the decay of the testis, and the pain and annoyance are really so great as to require something to be done to alleviate the patient's suffering;" (p. 474;) and these cases are certainly not of frequent occurrence. We may on this head notice an inconsistency in Sir A. Cooper's statements. In his large work on the testicle he says, "Varicocele should scarcely receive the title of a disease," but "in a *few* examples it produces uneasiness in the loins," (p. 219,) and "it does in a very rare case happen that persons suffer great pain in the testis and loins from a varicocele." (p. 223.) In the third vol. of Guy's Hospital Reports, however, he tells us "I have seen, in the course of my practice, *many persons* suffer so severely in body and in mind from this complaint, that they would readily submit to any operation which was not attended with danger to life to obtain relief." (p.7.)

Sir A. Cooper says that tying the spermatic vein is an operation which "he would dread most exceedingly," and he proposed the removal of a portion of the scrotum as a means of support, and diminishing the volume of the enlarged veins: this operation he had never performed at the time he proposed it, but he subsequently published in the work last quoted from, five cases of cure or relief effected by its means. With respect to this operation Mr. Curling informs us that he examined a man on whom Sir A. Cooper himself performed it, "but who derived so little benefit from the operation that he afterwards submitted to castration; and a medical friend lately informed me that in one of the published cases of success the disease subsequently returned as bad as ever." (p. 458.) And a good deal of inquiry has led Mr. Curling to conclude "that excision of a portion of the scrotum is calculated to arrest the progress of varicocele, and afford full and permanent relief, only in those cases in which the painful symptoms of the disease admit of being temporarily but completely removed by suspending the parts in the hand, or in a well-adjusted suspensory bandage." (p. 460.)

We need not follow Mr. Curling through his full and clear description of the various methods proposed for the radical cure of varicocele, by division of the vessels, by ligature, whether in the old way, or variously modified in its application by Davat, Ricord, &c., by compression, or by excision of the vessels. We shall merely notice two of the numerous plans described.

The first of these methods is one attributed to Mr. Luke. A ligature is carried through the root of the scrotum between the varicose mass of veins and the vas deferens, and the ends attached to an instrument termed a fistula tourniquet, by which the ligature can be tightened or loosened at pleasure. The ligature is drawn moderately tight at first, and as it becomes relaxed by cutting through the included parts, the pressure is kept up by turning the screw until the patient complains of pain. Mr. Curling, if driven to an operation, would prefer this plan, an important advantage of which, he thinks, "is the effect of the gentle pressure first produced in exciting sufficient irritation to cause obliteration of the veins before the constriction afterwards made by tightening the ligature cuts the vessel through." (p. 476.) Now this plan only belongs to Mr. Luke so far as the use of the screw tourniquet is concerned, for otherwise it is absolutely identical with that of M. Reynaud (de Toulon), the whole difference being that the French surgeon instead of employing a screw, tied his ligature with a slip-knot on a roll of diachylon plaster or a pledget of lint, and thus was enabled to regulate the amount of pressure. The screw tourniquet may, for aught we know, be a more commodious method of managing the ligature, but it, at the utmost, constitutes an improvement in the mechanical details of M. Reynaud's operation and should only have been mentioned as such, whereas M. Reynaud's name is not alluded to at all. The second method we have to notice originated thus:

"A surgeon suffering from a varix in the leg, having heard Sir C. Bell state in illustration of the fact of the dilatation of a varicose vein being caused solely by the pressure of the column of blood, that if the distended vein be compressed with the finger the swollen condition of the vessel beneath shortly disappears, was led to apply the principle thus indicated to the treatment of his own case, which was attended with a satisfactory result. This gentleman mentioned the circumstance to Mr. Aston Key, who was accordingly induced to adopt the same principle in the treatment of cases of varicocele, and in a private communication with which I have been kindly favoured by Mr. Key, he has assured me that he believes the practice resorted to is effective, and applicable to the majority of cases of varicocele." (p. 469.)

Mr. Curling goes on to observe that if in a case of varicocele pretty firm pressure be made on the spermatic cord, the veins, if previously emptied while the patient was lying down, will not fill again, or if distended will gradually be emptied in consequence of being relieved of the superincumbent column of blood. This, he remarks, is not incompatible with the well-known method of distinguishing a variocele from a hernia, as gentle pressure, for an obvious reason, will cause the veins to swell below. Mr. Curling has not noticed, what is worthy of remark, that Sir A. Cooper observed this very phenomenon, but gave it a wrong interpretation, for he says, in speaking of the diagnosis of this affection from hernia, (Guy's Hospital Reports, vol. iii, p. 5,) "but the pressure must not be sufficient to arrest the blood in the spermatic artery, or the veins will remain empty;" and looking to the mobility of the spermatic artery and the nature of the tissues by which it is surrounded in a case of varicocele, such as could even remotely simulate hernia, it will, we suppose, be readily admitted that the empty state of the veins observed by Sir A. Cooper, when too great pressure was exerted, arose, not from the artery being commanded, but, from the dilated veins being thoroughly compressed. These considerations have led to the proposal of treating varicocele by the application of a truss to the external abdominal ring, a proceeding which, it has hitherto been supposed, to use the words of Sir A. Cooper, "not only cannot benefit, but on the contrary, must necessarily have a pernicious influence, by preventing the free return of blood from the testis," (on the Testis, pt. ii, p. 221;) but the whole principle of the proposed method depends on the *degree of pressure* exerted by the truss.

"The object then of this method of treatment may be stated to be the maintenance, whilst the patient is in the upright position, of such a degree of pressure on the spermatic veins as may be sufficient to relieve them from the superincumbent weight of the blood, without at the same time endangering the integrity of the testis by obstructing the spermatic artery, and without causing so much uneasiness as to render the remedy as painful as, or more difficult to be borne than the disease. This pressure must be continued a sufficient time to enable the coats of the vessels to return to their natural dimensions, and to acquire strength to carry on the circulation. When this is effected the patient is cured." (pp. 470-1.)

The principle on which this plan of treatment is founded is unexceptionable, but it can hardly be called new; it has long been applied to the treatment of varix of the lower extremity, and has frequently succeeded, though it too often fails solely on account of the practical difficulty of carrying it into effect. The use of the thigh-truss in varix of the saphena vein is strictly analogous to the proposed treatment for varicocele. Mr. Curling is therefore quite right in saying "that the main difficulty of this treatment consists in the application of continuous local pressure." (p. 471.) He has heard of two cases in which the compressing instrument could not be borne, but in a third, Evans's patent truss effected a cure without producing the slightest inconvenience. If the mechanical difficulty can be surmounted, we certainly are inclined to anticipate that this mode of treatment will often be found effectual.*

The Fourth and last Part is devoted to the diseases of the scrotum. We regret that our exhausted space forbids our giving any account of these. We must, however, direct the reader's attention to the excellent account given of the *chimney-sweeper's cancer*, in chap. x, which contains several new and interesting particulars well worthy of notice. As the last part occupies little more than fifty pages, it will be seen that we have submitted to our readers a tolerably complete view of the work. The great length to which this article has extended indicates the very favorable opinion we have formed of Mr. Curling's book; for we certainly would not have allowed so much space to an inferior production. The author has not only been happy in the selection of his subject, as a work embracing within a moderate compass all the diseases of the testis and of the scrotum was very

* We learn from Mr. Curling, that since the publication of his work on the testis, he has treated three cases of painful varicocele by compression of the vessels applied with the lever-truss. In all three, very decided relief was afforded by this plan, and the patients were able, after a short time, to tolerate the necessary pressure without inconvenience. One, a copper-smith, has since been able to follow his laborious occupation, which he had been compelled to forego; and another, a medical assistant, to continue his duties in a standing posture for many hours of the day, without suffering from the disease. Equal benefit has resulted in a fourth case treated in this manner, by a medical friend of Mr. Curling's.

much wanting, but he has executed his undertaking with praiseworthy industry and zeal, aided by a sound practical judgment. The materials for the history of these diseases lay scattered in numerous journals, systematic works and detached treatises; Mr. Curling has collected and arranged them, so that they are now accessible in a single volume, and this he has done not as a mere compiler, but in a way that proves him to possess a thorough practical acquaintance with the matters in hand. We cannot in justice say that the author has thrown much new light on the nature and treatment of the disorders of the testicle, but we must equally in justice say that he has produced a work on those diseases superior to any yet published, and which we imagine is not likely to be soon superseded by a better. The volume is elegantly printed, and the woodcut illustrations by Bagg are beautiful.

---

